# The POU4F2/Brn-3b transcription factor is required for the hypertrophic response to angiotensin II in the heart

**DOI:** 10.1038/s41419-019-1848-y

**Published:** 2019-08-14

**Authors:** Laura Mele, Lauren J. Maskell, Daniel J. Stuckey, James E. Clark, Richard J. Heads, Vishwanie S. Budhram-Mahadeo

**Affiliations:** 1 Molecular Biology Development and Disease, UCL Institute of Cardiovascular Science, London, UK; 2 Centre for Advanced Biomedical Imaging (CABI), Division of Medicine, UCL Faculty of Medical Sciences, London, UK; 3 School of Cardiovascular Medicine and Sciences, Faculty of Life Sciences and Medicine, King’s College, London, UK

**Keywords:** Molecular biology, Cardiovascular diseases

## Abstract

Adult hearts respond to increased workload such as prolonged stress or injury, by undergoing hypertrophic growth. During this process, the early adaptive responses are important for maintaining cardiac output whereas at later stages, pathological responses such as cardiomyocyte apoptosis and fibrosis cause adverse remodelling, that can progress to heart failure. Yet the factors that control transition from adaptive responses to pathological remodelling in the heart are not well understood. Here we describe the POU4F2/Brn-3b transcription factor (TF) as a novel regulator of adaptive hypertrophic responses in adult hearts since Brn-3b mRNA and protein are increased in angiotensin-II (AngII) treated mouse hearts with concomitant hypertrophic changes [increased heart weight:body weight (HW:BW) ratio]. These effects occur specifically in cardiomyocytes because Brn-3b expression is increased in AngII-treated primary cultures of neonatal rat ventricular myocytes (NRVM) or foetal heart-derived H9c2 cells, which undergo characteristic sarcomeric re-organisation seen in hypertrophic myocytes and express hypertrophic markers, ANP/βMHC. The Brn-3b promoter is activated by known hypertrophic signalling pathways e.g. p42/p44 mitogen-activated protein kinase (MAPK/ERK1/2) or calcineurin (via NFAT). Brn-3b target genes, e.g. cyclin D1, GLUT4 and Bax, are increased at different stages following AngII treatment, supporting distinct roles in cardiac responses to stress. Furthermore, hearts from male Brn-3b KO mutant mice display contractile dysfunction at baseline but also attenuated hypertrophic responses to AngII treatment. Hearts from AngII-treated male Brn-3b KO mice develop further contractile dysfunction linked to extensive fibrosis/remodelling. Moreover, known Brn-3b target genes, e.g. GLUT4, are reduced in AngII-treated Brn-3b KO hearts, suggesting that Brn-3b and its target genes are important in driving adaptive hypertrophic responses in stressed heart.

## Introduction

Terminally differentiated cardiomyocytes in adult hearts respond to increased workload or chronic haemodynamic stress by undergoing hypertrophy. During early adaptive responses, cardiomyocyte undergo characteristic changes including increased size, cytoskeletal re-organisation and metabolic switching, which are necessary for maintaining cardiac output^1,2^. Under physiological conditions, e.g. endurance exercise or pregnancy, such hypertrophic changes can be reversible^3^. In contrast, sustained pathological stresses, which can be induced by different stressors including neurohumoral activation (e.g. AngII), volume/pressure overload (e.g. hypertension and aortic stenosis), or acute cardiac injury (e.g. myocardial infarction), can drive irreversible, pathological changes such as cardiomyocyte apoptosis, interstitial fibrosis and adverse remodelling that precede progression to heart failure^4,5^.

Changes in the hypertrophic heart arise due to alterations in gene expression following the activation of well-characterised signalling pathways^6–11^. For example, the vasoactive peptide, AngII, acts as a Gα(q)-coupled receptor agonist to activate different kinase pathways e.g. mitogen-activated protein kinase/extracellular signal-regulated kinase1/2 (MAPK/ERK); c-Jun N-terminal kinase or p38 MAPK^12,13^, which in turn, converge on key master regulators to control gene expression^6–11^. Similarly, the calcium-dependent protein phosphatase, calcineurin A (CnA), activates the nuclear factor of activated T-cells (NFAT) transcription factor (TF), which regulates hypertrophic genes^11,13,14^.

Gene expression changes can significantly affect cardiomyocyte fate and function. For example, early hypertrophic responses are associated with re-expression of foetal genes [e.g. atrial natriuretic peptide (ANP), β-myosin heavy chain (β-MHC)]; cell cycle regulators (e.g. cyclin D)^15–17^; activation of oncogenes (e.g. c-myc and c-fos)^18,19^ and increased insulin-responsive glucose transporter GLUT4^20^. On the other hand, chronic stress or injury, which induces pro-apoptotic genes such as p53 and Bax, will drive irreversible cardiomyocyte apoptosis, interstitial fibrosis and heart failure^21–25^.

Therefore, gene expression must be tightly regulated at multiple levels and central to this process is gene transcription by the RNA polymerase II (RNAPII) enzyme. RNAPII-mediated gene transcription depends upon DNA-binding TFs, which promote the assembly of the transcriptional complex and rate of transcription^26^. Tissue-specific TF, which are expressed in specific cells or under different conditions, often control multiple target genes and therefore act as master regulators that determine cell fate. In the heart, TFs such as GATA4, MEF2 and NKX2.5 are implicated in transcription of cardiac genes under different conditions^11,27,28^. We are studying the POU4F2/Brn-3b TF, (called Brn-3b), which was originally isolated from neuronal cells but has since been identified in many other tissues including the heart^29–31^. Brn-3b is a member of the POU (Pit-1/Oct-1/Unc-86) homeodomain TF family, characterised by a highly conserved POU DNA binding domain. The gene encoding Brn-3b, consists of two exons separated by an intron, which can give rise to two distinct isoforms of Brn-3b, via alternative promoter usage. The longer Brn-3b(l) isoform is encoded by both exons 1 and 2, whereas the shorter Brn-3b(s), is encoded by exon 2 only. At present, the roles of different Brn-3b isoforms are unclear, but an auto-regulatory feedback loop links expression of the different isoforms^29^.

Importantly, Brn-3b controls transcription of multiple target genes, but its effects are highly dependent on the tissue of expression and growth conditions^29–31^. For example, Brn-3b is essential for survival and function of retinal ganglion cells (RGCs) in the eye because constitutive Brn-3b knockout (KO) mice develop progressive blindness due to loss of RGCs. In these cells, Brn-3b target genes include Pax4 and sonic hedgehog^32,33^. However, Brn-3b promotes cell proliferation/growth in other cells by activating cell cycle genes, cyclin D1/CDK4^34–37^ but can confer drug resistance and migratory potential to cancer cells by activating the small heat shock protein, HSP27 and/or repressing adhesion molecule plakoglobin (γ-catenin)^38,39^. Yet, if Brn-3b is co-expressed with the growth inhibitory p53 protein, then it cooperates with p53 to maximally increase transcription of pro-apoptotic genes such as Bax, thereby increasing apoptosis^40–42^. Therefore, to understand its mechanism of action, it is important to identify tissue-specific effects of Brn-3b.

Brn-3b is highly expressed in foetal hearts with expression detected in cardiomyocytes in heart sections and primary isolated cultures of cardiomyocytes^43^. Although expressed at lower levels in adult hearts^42^, Brn-3b is increased throughout the myocardium by 24 h after coronary artery ligation^40^. Around the infarct zone, Brn-3b co-expression with p53 causes increases Bax protein linked to cardiomyocyte apoptosis. Brn-3b is required for maximal Bax induction when co-expressed with p53 in injured cardiomyocytes because reducing Brn-3b in primary neonatal rat ventricular cardiomyocytes (NRVM) cultures, using short interfering RNA (shRNA) blocks Bax expression, even when p53 expression is intact^40,42^. However, the effects of Brn-3b in non-injured myocardium, lacking p53 is still unknown and must be investigated.

In this study, we demonstrate that known hypertrophic mediators, AngII and CnA, increase Brn-3b in intact mouse hearts, primary NRVM cultures and H9C2 cells. Differential regulation of known Brn-3b target genes following AngII treatment indicate potential roles in controlling cell fate. In vivo studies identified baseline differences in contractile efficiency in male Brn-3b KO hearts while attenuated hypertrophic response to AngII in male hearts, is linked to extensive fibrosis and adverse re-modelling, suggesting that mutant hearts are unable to adapt to stress.

## Materials and methods

### Materials

General laboratory reagents: Merck (Nottingham, UK) and Sigma (Dorset, UK), unless otherwise stated. Primary antibodies: Brn-3b-rabbit pAb (Abcam-Cambridge, UK); mouse β-MHC Ab (Gene Tex, CA, USA); α-actinin mAb (Sigma, UK): GLUT4 (Cell Signalling Technology, USA), GAPDH and cyclin D1 Ab (Santa Cruz, California, USA) and β-tubulin (Cell Signalling Technology, USA), p53 pAb (Calbiochem); anti-rabbit Bax pAb (Pharmingen); rabbit anti-phospho-SMAD 3 pAb (Millipore). (HRP-conjugated secondary Ab was from Dako (Cambridgeshire, UK). MEK inhibitor, PD98059, p38 kinase inhibitor: SB203580, CnA inhibitors: cyclosporine A (CsA) and ascomicin were from Calbiochem (Nottingham, UK). H9c2 cell line was obtained from ATCC (Manassas, VA, USA) and hearts from calcineurin Aα transgenic mice were a kind gift from Prof. J Molkentin (Children’s Hospital Medical Center, Cincinnati Ohio, USA). Primer Sequence for RT-PCR:

ACTB: F-5′GGCTGTATTCCCCTCCATCG 3′; ACTB R- 5′CCAGTTGGTAACAATGCCATGT 3′

ANF: F-5′ TGGGCTCCTTCTCCATCACC 3′; ANF R - 5′ GCCAAAAGGCCAGGAAGAGG 3′

β-MHC F-5′GCCTACCTCATGGGACTGAA 3′; β-MHC R - 5′ ACATTCTGCCCTTTGGTGAC 3′

Brn-3b F-5′ GAGAGAGCGCTCACAATTCC 3′; Brn-3b R- 5’ ATGGTGGTGGTGGCTCTTAC 3′

Bax F-5′ CTGCAGAGGATGATTGCTGA 3′; Bax R 5′ GATCAGCTCGGGCACTTTAG 3′

B2M F-5′ GGTCTTTCTGGTGCTTGTCTCA 3′; B2M R 5′ GTTCGGCTTCCCATTCTCC 3′

GAPDH F-5′ CTTCATTGACCTCAACTAC 3′; GAPDH R 5′ AGTGATGGCATGGACTGTG 3′

## Methods

### In vivo AngII treatment and echocardiography

All animal experiments were carried out in compliance with Home Office regulations (Animals Scientific Procedures Act 1986) and were approved by local UCL Ethics Review Board. Early studies were undertaken using wild-type (WT) C57Bl/6 mice purchased from commercial companies (Harlan, UK) and at later stages, using KO mice and age/sex matched WT littermate controls obtained by crossing inbred Brn-3b heterozygotes. For early studies, 2-month-old mice (purchased from Harlan UK) were anaesthetised [1.5% isoflurane v/v in 100% O_2_ with analgesia (buprenorphine 0.1 mg kg^−1^ SC)] and subcutaneous implantation of Alzet osmotic mini pumps (at the nape of the neck) used to administer AngII (4.5 mg/kg/day) for up to 4 weeks. Saline was given to age-matched control groups.

To assess for changes in cardiac function and heart dimensions in Brn-3b KO mutants and age-matched WT controls, echocardiography was undertaken in anaesthetised mice on a weekly basis, using high frequency (30 MHz) ultrasound system (Visualsonics Vevo 2100, Toronto, CA, USA)^44^. At the end of studies, measurement of the heart weight and body weight (HW:BW) ratio was combined with parameters such as left ventricular (LV) mass and septal thickness to determine hypertrophic changes^45^. Since the study was intended to analyse gene expression and effects specifically in hypertrophic hearts, then key hypertrophic endpoints, e.g. increased LV mass or HW:BW ratio, were used to identify outliers, i.e. WT mice that showed no hypertrophic responses to AngII treatment possibly because of blockages in the mini osmotic pump.

At the end of experiments, isolated hearts were either snap-frozen for RNA or protein extraction or fixed in 4%PFA and subsequently paraffin embedded for sectioning. Statistical analysis was carried out using Students’ *t* test to identify significant changes (*p < 0.05) between two groups whilst two-way ANOVA and Bonferroni post-hoc test were carried out to identify differences within multiple groups.

### Pressure–volume measurements

Assessment of pressure–volume relationship in mouse hearts were achieved using the Admittance-derived PV loop system (ADVantageTM; Scisense Inc.), as previously described^45,46^. Briefly, mice were anaesthetised with isoflurane by passive inhalation then intubated for continued administration of anaesthetic (1.5% v/w) in 100% oxygen using a ventilator (Hugo Sachs electronic MiniVent type 845, Germany). Body temperature was monitored using a rectal thermometer and maintained around ~37 °C, throughout the experiment by using a thermostatically controlled heating pad. An ultra-miniature conductance catheter (Scisense Inc., London, Canada) was inserted into the left ventricle via the apex, as previously described^46^ and connected to the ADVantage™ system for generation of PV loops, which were recorded using the PowerLab multi-channel acquisition system (Powerlab, ADInstruments, UK). PV loops were acquired at baseline followed by altered preload, achieved using transient (4–5 s) occlusion of the inferior vena cava. Data collected using PowerLab software were analysed for significant changes using appropriate statistical packages e.g. Excel or Prism. Statistical significance, indicated by * or **, was determined by student’s *t* test and defined as *p* < 0.05 or <0.01 respectively.

### Primary cultures

Primary NRVM cultures were prepared from hearts of 1–2-day-old Sprague Dawley rats as previously described^40^. Briefly, isolated neonatal hearts were washed in ice-cold ADS buffer (116 mM NaCl, 20 mM HEPES, 0.8 mM NaH_2_PO_4_, 5.6 mM glucose, 5.4 mM KCl, and 0.6 mM MgSO_4_) pH 7.35, cut into pieces and digested with Type 2 collagenase (0.5 mg/ml) (Worthington Biochemical Corp, NJ) and pancreatin (0.6 mg/ml) (Sigma). The cells were pelleted by centrifugation then resuspended in Dulbecco’s Modified Eagle’s Medium (DMEM): Medium 199 (4:1) containing 10% horse serum and 5% foetal calf serum (FCS) (Gibco, Invitrogen Corporation, Auckland, NZ). Following pre-plating for 1h to remove fibroblasts, cells were plated on gelatine-coated six-well plates (2 × 10^6^ cells/well). After 24 h, media were replaced with 4:1 DMEM/Medium 199 supplemented +1% FCS and incubated for 48 h before experiments. Cells were treated with AngII (at final concentrations of 10 µg/ml or 30 µg/ml) and incubated for the times indicated.

### Cell culture, transfection and luciferase assay

H9c2 cells, grown in DMEM + 10% FCS + 1% pen/strep, were plated onto six-well plates (5 × 10^5^/well) and treated as specified e.g. AngII (10 µg/ml); PD98059 (10-20 µm); CsA (1 µm). Ascomicin **(**1 µm) pretreatment was for 30 min prior to adding AngII for 24 h. For transfection studies, GeneJuice^TM^ (Merck Biosciences, Nottingham, UK) was used according to the manufacturer’s protocol co-transfected the Brn-3b reporter construct (Brn-3b promoter driving expression of the luciferase reporter) and expression vector encoding CnA. Unless otherwise specified, cells were harvested after 48 h into lysis buffer and the extracts were used to analyse for changes in promoter activity. Briefly, 20–50 µL of extract was used to measure luciferase activity using the TD 20/20 luminometer (Turner Designs, Promega, Southampton, UK) and the Dual-Luciferase Reporter Assay System (Promega, Southampton, UK), according to the manufacturer’s protocol. Values for firefly luciferase reporter activity were adjusted using renilla luciferase controls to correct for transfection efficiency then expressed as percentage of vector control. Statistical analyses were carried out using students *t* -test and significance *p < 0.05.

### RNA extraction, cDNA synthesis and quantitative polymerase chain reaction (qRT-PCR)

RNA was isolated from snap-frozen, homogenised hearts using TRIZOL^®^ Reagent (Invitrogen), whereas NRVM or H9c2 cells were harvested in Trizol then processed according to the manufacturer’s protocol. Contaminating genomic DNA was removed from RNA by treatment with RNAse-free DNAse1 (Promega, Southampton, UK) and 1 μg of total RNA was used for cDNA synthesis (Superscript™ II Reverse Transcriptase (Invitrogen)). Quantitative reverse transcriptase (RT) polymerase chain reaction (qRT-PCR) was carried out using the Opticon 2 DNA engine thermal cycler (BioRad) and Taqman probes (ABI) or SYBR Green chemistry with specific primers for each gene. Housekeeping genes, beta-2 microglobulin (B2M) and GAPDH were used to correct for variability between samples. Statistical analysis was carried out using student’s *t* test. Significance is shown as *p < 0.05.

### Protein preparation and quantification

Cells were harvested in Laemmli buffer; mouse hearts were homogenised in liquid nitrogen then resuspended in Laemmli buffer +10% (v/v) β-mercaptoethanol; boiled (100 °C, 5 min) and centrifuged (13 × *g*, 10 min RT) to remove cellular debris. Total cellular proteins were resolved by polyacrylamide gel electrophoresis (10–12.5% gels) and western blot analysis carried out as previously described^40^. Briefly, block buffer [phosphate-buffered saline + 0.1% Tween-20 (PBS-T); 4% milk] was incubated for 1 h; primary Ab was incubated for 1 h (RT) or overnight (4 °C). After five washes (5 min; RT; PBS-T + 0.1% milk) secondary Ab was incubated for 1 h then washed (4 × 5 min; RT; PBS-T + 0.1% milk and final 1 × 5 min wash in PBS-T), signals were developed using enhanced chemiluminescence reagent (Bio Rad, UK). Immunoblots were analysed using densitometry or image J.

### Co-immunostaining for protein localisation

Cultured cells grown on coverslips were either treated with AngII or left untreated (control) (as specified) then fixed (4% PFA; 15 min), washed in 1× TBS and incubated in block solution [20% goat serum in TBS +0.1% triton-X100 (TBST)] for 60 min. Primary antibodies [rabbit Brn-3b pAb (Abcam-1:500) and α-actinin mAb (Sigma, UK)] were incubated for 1 h at RT or overnight at 4 °C. If sequential co-immunostaining was carried out, the second primary antibody was incubated after washing cells (×5 with TBST; 1 h; RT). Second Ab (was appropriate fluorescent conjugated) was incubated for 45 min to 1 h, RT and following washes, cells mounted in mounting medium containing DAPI (Vector Laboratories) and imaged using the Zeiss Axioscop 2 fluorescent microscope and analysed with Axiovision software or Image J.

### Chromatin Immunoprecipitation (ChIP) assay

This technique was carried out as described by Lee et al. and Ounzain et al.^38,47^ using rat heart-derived H9C2 cells treated with 10 µg/ml AngII for 24 h or untreated for control. Anti-NFAT Ab (BioLegend) was used to immuneprecipitate NFAT proteins bound to the Brn-3b promoter, whereas anti-β-tubulin or 2nd Ab (Dako) was used for negative control ChIP samples. The positive control DNA, Input, was removed from the samples after sonication but prior to immunoprecipitation with Abs. Input or ChIP DNA were used for PCR with multiple primer sets that flanked putative NFAT sites in the Brn-3b promoter, and bands shown were obtained following PCR using the following primer set to amplify ChIP DNA or positive/negative control DNA. Forward primer 5′ GAAGTGAAAC TCTGACTTGAC 3′; Reverse primer 5′ CAGCTCTGAGATATTTTTAGTG 3′. PCR products were resolved by gel electrophoresis (2% agarose TBE gel) and imaged using a CCD camera.

### Immunohistochemistry of paraffin-embedded heart sections

Heart sections were dewaxed and rehydrated before antigen retrieval [microwave for 10 min in 0.01 M sodium citrate (pH 6.0)] and incubation with 0.3% hydrogen peroxide (30 min, RT). Sections were incubated with the blocking solution i.e PBST [(phosphate-buffered saline + 0.1% Tween 20) +(10% goat serum)] for 30 min, RT followed by incubation with primary antibody [e.g. rabbit anti-phospho-SMAD 3 pAb° (Millipore, 1:50)] in a humidified chamber; overnight at 4 °C. Negative secondary Ab° control was included in each experiment. After washes (5 × 5 min) with PBST, slides were incubated for 1 h (RT) with the biotinylated anti-rabbit secondary (Cell Signalling 1:200), and colorimetric detection was carried out using DAB substrate (Sigma), according to the manufacturer’s protocol. After immunostaining, slides were dehydrated in graded ethanol series, washed twice in Xylene before mounting. Slides were imaged using the Hamamatsu Nanozoomer whole-slide imaging function and analysed using the NDP view 2 software (Hamamatsu, Japan).

### Masson’s trichrome staining

This technique was used to analyse for changes in cardiac morphology and assess fibrosis (ECM deposition). Formalin fixed, paraffin embedded heart sections from Brn-3b KO and WT mice were dewaxed, rehydrated then stained with Masson’s trichrome, using the SAKURA Tissue Tek DRS 2000 automatic slide stainer (Sakura Finetek Europe, Netherlands). Slides were cover slipped on an automated Glass Coverslipper (e.g. Leica CV5030), imaged using Hamamatsu Nanozoom whole-slide imaging function and analysed using the NDP view 2 software (Hamamatsu, Japan).

### Assessment of cardiomyocyte size using wheat germ agglutinin (WGA) staining

WGA staining was used to assess changes in the cardiomyocyte size in WT and Brn-3b KO hearts following AngII treatment when compared with saline control hearts. Following dewaxing and rehydration, heart sections were incubated with 5 µg/ml WGA 488A conjugate (Biotium) in HBSS in the dark (30 min at RT). Unbound WGA was washed with HBSS (×1) and PBS (×2) and sections were mounted using mounting medium containing DAPI. Slides were imaged using AxioScan.Z1 and cell area was analysed using ImageJ software with >30 cells measured for each heart section.

### TUNEL staining

To evaluate cell death in heart sections, TUNEL staining was undertaken using the DeadEnd Colorimetric TUNEL System (Promega, Southampton, UK) according to the manufacturer’s protocol. Sections were imaged using Hamamatsu Nanozoomer whole-slide imaging function and analysed using the NDP view 2 software (Hamamatsu, Japan).

## Results

### AngII stimulates Brn-3b expression in hypertrophic cardiomyocytes

**Fig. 1:**
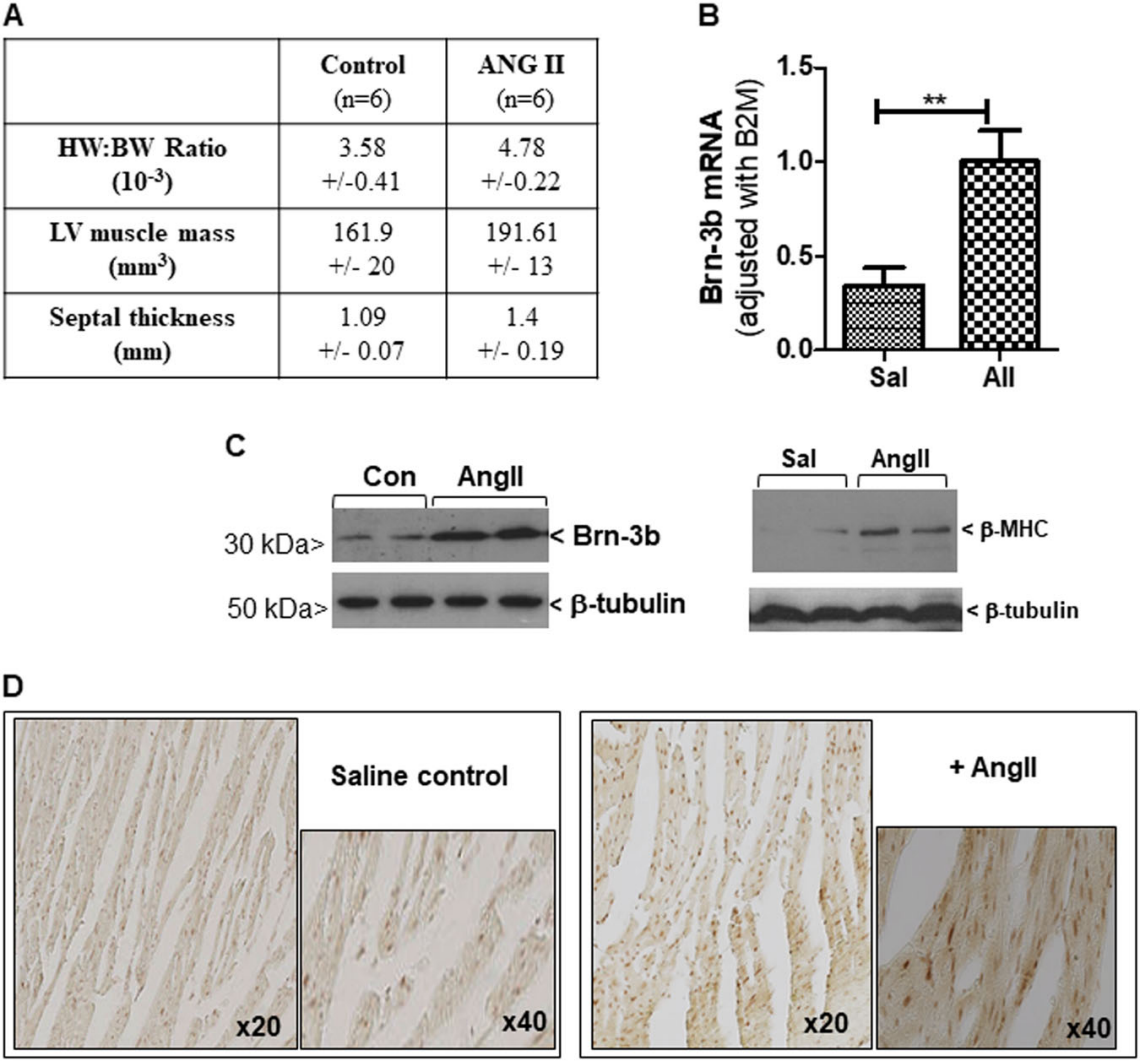
Altered Brn-3b expression mRNA in mouse hearts treated with AngII. **a** Assessment of hypertrophic responses in hearts taken from AngII-treated wild-type C57BL/6 mice and compared with saline-treated control mouse hearts. Measurements of heart weight:body weight ratio (HW:BW); LV mass and septal thickness is shown as mean and standard error from different experimental groups (*n* = 6). **b** Results of quantitative RT-PCR (qRT-PCR) showing increased Brn-3b mRNA in hearts taken from AngII-treated mice compared with saline controls. Statistical significance between groups (≥5 mice) (**p < 0.01) was analysed using students *t* test. **c** Representative western blot showing increased (i) Brn-3b or (ii) beta MHC proteins in mouse hearts either treated with AngII for up to 4 weeks, compared with saline control (Con). **d** Representative DAB immunostaining showing increased Brn-3b protein expression in AngII-treated mouse hearts. Images were taken using the Hamamatsu Nanozoomer scanner and shown at ×20 and ×40, as indicated

Since Brn-3b re-expression in injured adult hearts affects gene expression and cell fate^40^, we tested if this TF is increased in response to stimuli that induce hypertrophic responses in the heart. Therefore, Brn-3b levels were analysed in WT mouse hearts following treatment with AngII for up to 4 weeks and compared with saline infused control (Con) mouse hearts. Figure 1a shows that AngII treatment caused the expected hypertrophic changes including increased HW:BW ratio, LV mass and septal thickness compared with saline control hearts. AngII-treated hearts also expressed increased Brn-3b mRNA (Fig. 1b) whereas western blot analysis confirmed that similar increases in Brn-3b protein, correlated with induction of hypertrophic marker β-MHC (Fig. 1c). Increased immunostaining of Brn-3b in sections of AngII-treated hearts also confirmed its activation by AngII (Fig. 1d).

**Fig. 2:**
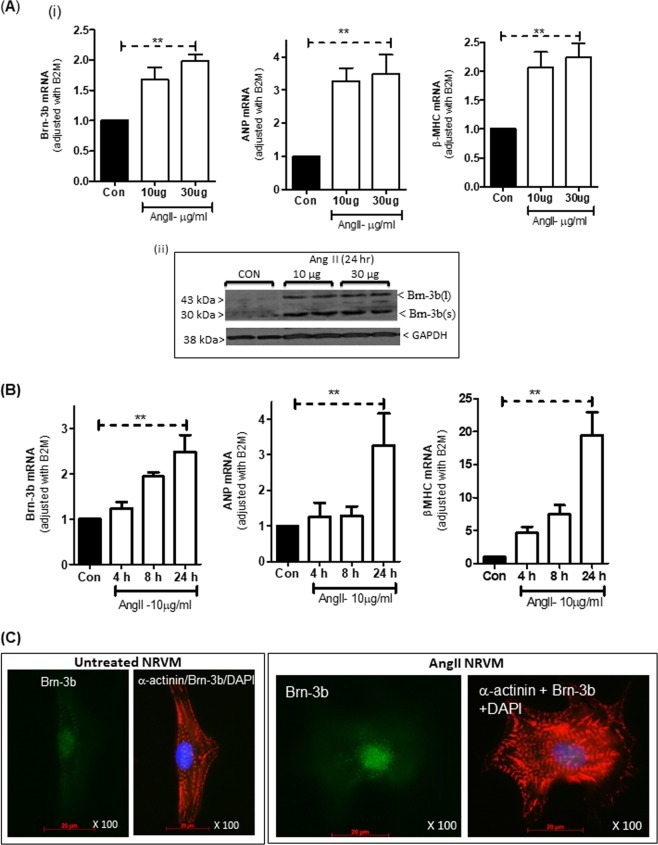
Brn-3b induction by AngII in neonatal rat ventricular myocytes (NRVM) cultures. (**a**) (i) qRT-PCR data showing changes in mRNA encoding Brn-3b or hypertrophic markers ANF and β-MHC in NRVM treated with different concentrations (10 or 30 µg/ml) of AngII for 24 h, when compared with untreated control cells (Con). Variations in RNA from different samples were standardised using the housekeeping gene, β2-micro-globulin (B2M) mRNA expression and values were expressed relative to untreated control cells set at 1. Graphs represent the mean values ± SD from independent NRVM samples (*n* = 6). *p < 0.05. (ii) Representative western blot analysis showing induction of Brn-3b proteins following treatment with 10 µg/ml AngII for 24 h. Different Brn-3b isoforms (~43 and ~32 kDa) are indicated and the invariant GAPDH protein indicated any variation in protein loading. (**b**) Quantification of mRNA encoding Brn-3b or hypertrophic markers, ANP and β-MHC in NRVM cultures treated with 10/ml AngII for different times (4, 8, 24 h). Variations in mRNA between samples were standardised using B2M and values expressed relative to untreated control cells set at 1. Graphs represent the mean values ± SD from six independent experiments (*n* = 6). **p < 0.01 compared with untreated controls (Con). (**c**) Representative immunofluorescent images showing localisation of Brn-3b protein (Alexa Fluor 488–green) in AngII-treated NRVM co-stained with phalloidin (red) to demonstrate actin cytoskeleton reorganisation and DAPI staining (blue) showing cell nuclei. Images shown were taken at ×100 magnification (oil immersion lens) using the Zeiss Axioskop 2 microscope

We next tested if AngII treatment affected Brn-3b levels in isolated cardiomyocytes by analysing its expression in primary NRVM cultures, treated with 10 or 30 µg/ml of AngII, for 24 h. Figure 2a(i) shows that 10 or 30 µg /ml AngII caused similar increases in Brn-3b mRNA, which correlated with induction of hypertrophic markers, ANP and βMHC. Western blot analysis showed increased protein in AngII-treated cells, particularly Brn-3b(s) isoform (Fig. 2a(ii)). Time-course studies demonstrated that treatment with 10 µg/ml AngII, which induced hypertrophic markers ANP and β-MHC, also increased Brn-3b mRNA after 8 or 24 h (Fig. 2b). Therefore, subsequent experiments were carried out using 10 µg/ml AngII treatment for 24 h.

Co-immunofluorescence staining was undertaken using Brn-3b and α-actinin antibodies to study Brn-3b localisation and cytoskeletal changes in AngII-treated or untreated NRVM cultures. Representative images (Fig. 2c) show nuclear localisation of Brn-3b in AngII-treated NRVM, which display characteristic cytoskeletal re-organisation of hypertrophic responses when compared with control cells.

**Fig. 3:**
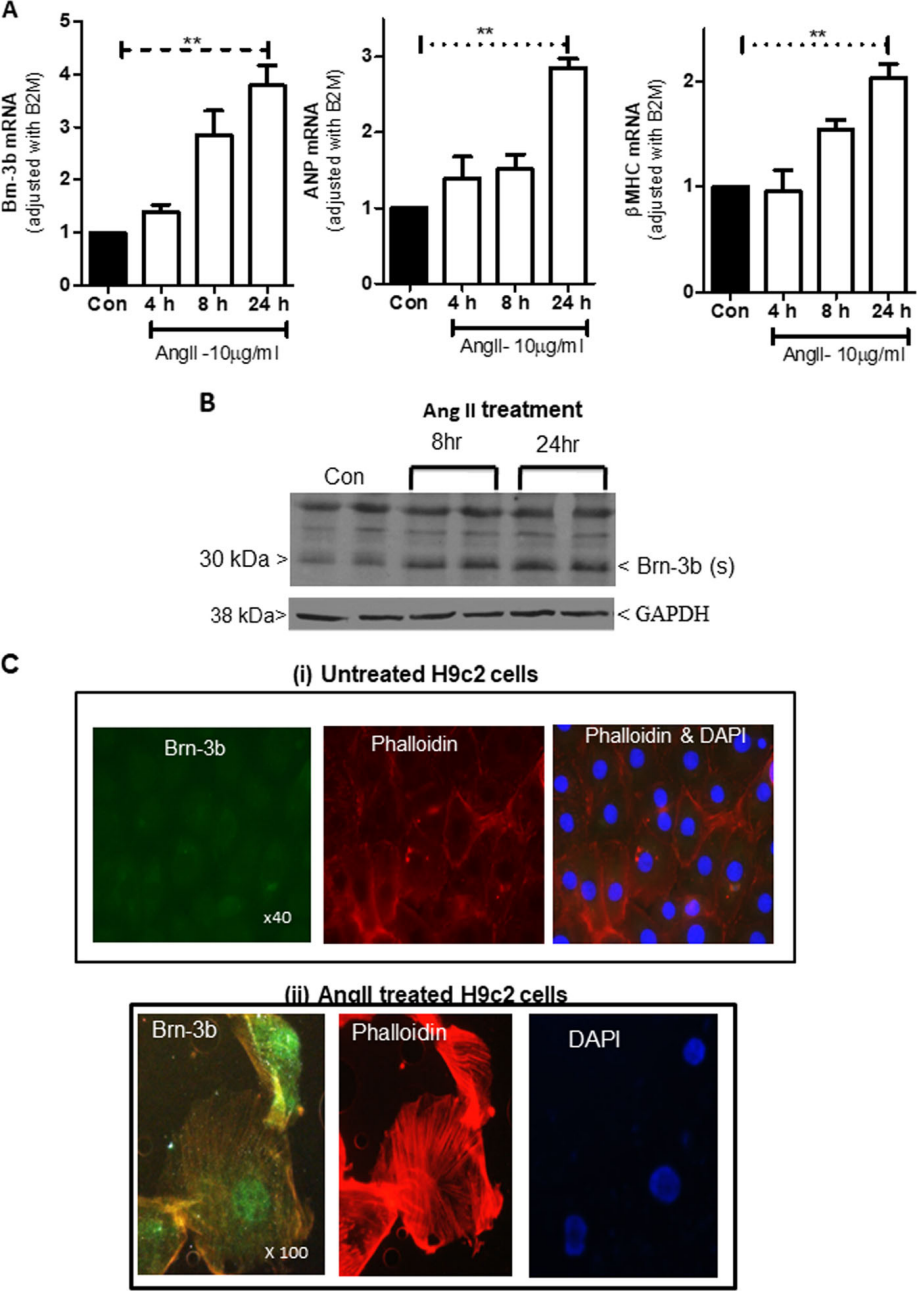
Brn-3b induction by AngII in H9c2 cell cultures. **a** qRT-PCR data showing changes in mRNA encoding Brn-3b, ANP and β-MHC in H9c2 cells treated with 10 µg/ml of AngII for 4, 8 or 2 h when compared with untreated controls (Con). Variation between RNA samples was standardised using B2M, and values are expressed relative to the untreated control cells set at 1. Data represent values from six independent experiments and **p < 0.01, where (*n* = 6). **b** Representative western blot analysis showing induction of Brn-3b(s) protein following treatment with 10 µg/ml AngII for 24 h and the invariant GAPDH protein indicates any variability in protein loading. **c** Representative immunofluorescent images showing Brn-3b protein localisation (green) in AngII-treated H9c2 cells stained with phalloidin (red) to show cytoskeletal re-modelling. DAPI staining (blue) shows the cell nuclei. Untreated control cells are shown at ×40 to demonstrate little protein expression in a larger field of view, whereas ×100 magnification of AngII-treated cells highlights nuclear Brn-3b expression in cells that display extensive cytoskeletal remodelling

Similar studies were conducted in the embryonic rat heart-derived H9c2 cells, which express Brn-3b^40^ and undergo characteristic hypertrophic changes in response to AngII treatment^48^. Figure 3a, b shows that AngII induced comparable increases in Brn-3b mRNA and protein levels while immunostaining confirmed nuclear localisation in AngII-treated cells (Fig. 3c). Since AngII induces Brn-3b in both NRVM and H9c2 cells, these results support H9c2 cell line as an appropriate model for in-depth studies to understand Brn-3b effects/role(s) in mediating hypertrophic responses.

### Brn-3b promoter is activated by known hypertrophic signalling pathways

**Fig. 4:**
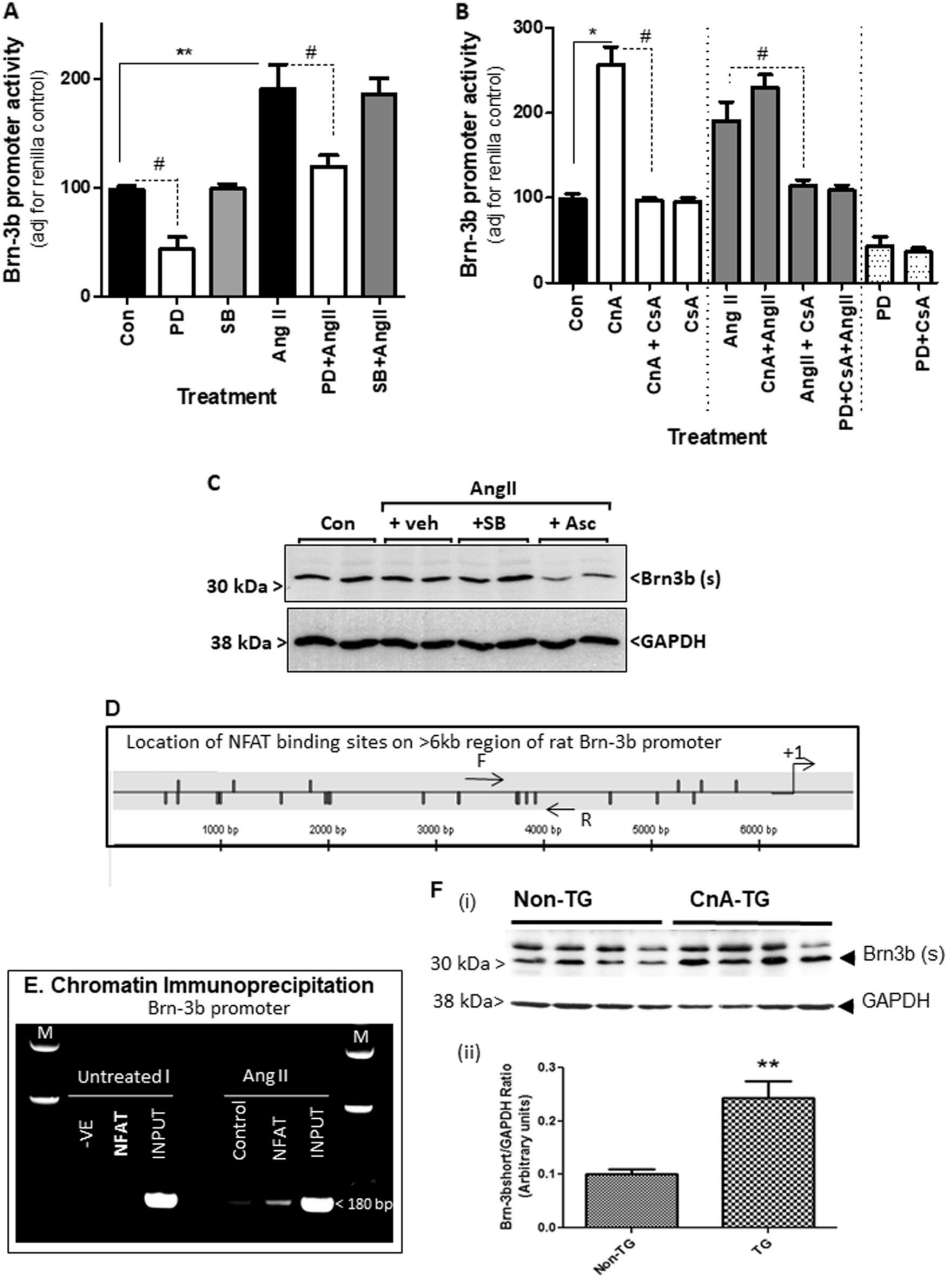
Signalling pathways involved in AngII activation of Brn-3b. **a** Results of reporter assays carried out using extracts from H9c2 cells transfected with Brn-3b promoter either alone (Con) or following treatment as specified i.e. angiotensin II (AngII) (10 µg/ml) ±MEK1/2 inhibitor (PD) at 20 µM (or ±p38 MAPK inhibitor (SB) at  5 µM. Promoter activity was expressed as % untreated control cells (transfected with reporter construct or empty expression vector), set arbitrarily at 100%. Data from six independent samples are shown and statistical significance (*p < 0.05 increase, ^#^*p* < 0.05 reduction) was determined using two-way ANOVA followed by post-hoc Bonferroni test. **b** Results of reporter gene assays showing changes in Brn-3b promoter activity in cells that co-express constitutively active CnA either alone (CnA) or with the calcineurin inhibitor, CsA (CnA + CsA), when compared with CsA alone (1 µM). Promoter activity are also shown under different combinations of treatment i.e. CnA + AngII, in the absence or presence of different inhibitors, CsA and/or PD. The data represent the results of independent experiments (*n* = 6) and are expressed relative to levels found in untreated control cells (set at 100). Significant increases: **p < 0.01, *p < 0.05; ^##^significant reduction following treatment with inhibitor CsA (1 µM). **c** Western blot analysis showing Brn-3b expression in H9c2 cells either untreated (Con) or pretreated as indicated with either MAPK/ERK inhibitor, SB203580 (SB:5 µM) or calcineurin inhibitor, ascomycin (Asc:1uM) for 30 min prior to AngII treatment (10 µg/ml). **d** Schematic diagram showing the location of multiple NFAT binding sites on 6 kb region of the rat Brn-3b promoter following bioinformatic analysis using MatInspector™. +1 indicates the approximate position of the putative start site. PCR primers were designed to flank putative NFAT binding sites in the Brn-3b promoter. Horizontal arrows indicate positions of forward (F) and reverse (R) primers which were well conserved between rat and human sequence and which were used for ChIP assay. **e** PCR products obtained using the indicated primers set flanking NFAT sites, indicated by arrows (above) input DNA (chromatin DNA prior to immunoprecipitation) or ChIP DNA obtained after immunoprecipitation with NFAT Abs in either untreated or AngII-treated H9c2 cells. This was compared with negative ChIP DNA control (incubated with second Ab) or PCR negative (without DNA). The marker lane (M) shows the DNA ladder used to identify fragment size in the gel. **f** (i) Representative western blot showing Brn-3b(s) protein in hearts isolated from 4-week transgenic (TG) mice with cardiac-specific CnA overexpression or non-transgenic (non TG) littermates. The invariant protein GAPDH was used to control for protein loading. (ii) Quantification of Brn-3b(s) proteins in WT or CnA-TG mouse hearts, following normalisation with control GAPDH protein. **p < 0.01

AngII-mediated hypertrophic responses in the heart can be elicited by activation of different signalling pathways including p42/44MAPK/ERK, which was shown to activate the Brn-3b promoter in cancer cells^47^. Therefore, we next tested if this pathway also activated the Brn-3b promoter in response to AngII treatment. For this, H9c2 cells were transfected with a Brn-3b reporter construct, in which the Brn-3b promoter drives expression of the firefly luciferase reporter gene. After 24 h, transfected cells were treated with MEK1/2 inhibitor, PD98059 or selective p38 inhibitor, SB203580, while controls were left untreated. Following AngII treatment for another 24 h, reporter assays were undertaken. Figure 4a shows that PD98059 repressed basal promoter activity, whereas the SB203580 had no effect on basal promoter activity. As expected, AngII treatment activated the Brn-3b promoter and its effects were blocked by treatment with PD98059 but not SB203580, indicating that AngII activation of Brn-3b was mediated via p42/44MAPK/ERK1 pathway.

We next tested if CnA also activated Brn-3b promoter by co-transfecting the Brn-3b reporter construct with constitutively active CnA expression vector. After 24 h, transfected cells were treated with a CnA inhibitor, CsA for 24 or left untreated (control). Reporter assays showed that CnA activated the Brn-3b promoter when compared with vector control (Fig. 4b). CsA did not affect basal promoter activity but blocked induction by CnA, suggesting direct activation of the Brn-3b promoter by CnA. AngII treatment of CnA-transfected cells did not increase Brn-3b promoter activity further but treatment with CsA prevented AngII-mediated promoter activation. Combinations of CsA and PD98059 had no further effect on promoter activity. This data suggests crosstalk between CnA and MAPK/ERK pathways in activating the Brn-3b promoter in hypertrophic cells, with potential convergence of these pathways downstream of ATR1 activation^49^. This was confirmed in separate studies showing that the FK506 analogue, ascomicin, which is another CnA inhibitor, also reduced Brn-3b protein expression in AngII-treated H9c2 cells (Fig. 4c).

Since CnA normally mediates its effects by activating NFAT TFs^14^, bioinformatic analysis was done on the 6 kb proximal Brn-3b regulatory promoter sequence. This approach identified multiple NFAT-binding sites throughout the Brn-3b promoter (Fig. 4c). Therefore, ChIP assays were performed to test for NFAT binding to the Brn-3b promoter, in vivo, in untreated or AngII-treated H9c2 cells. ChIP DNA obtained using NFAT antibody, positive control (input DNA) or negative control (unrelated Ab) was used for PCR with primers designed to flank NFAT sites, across the promoter. Figure 4e shows the PCR products (~180 bp) obtained following amplification with the primers shown in Fig. 4c, using input DNA (positive control) or ChIP DNA immunoprecipitated from AngII-treated cells with NFAT Ab but not in untreated cells or Ab control. These results confirm that NFAT indeed binds to the Brn-3b promoter in hypertrophic cells while enhanced binding following AngII treatment supports activation of CnA/NFAT by AngII and a direct role in regulating Brn-3b expression.

To determine if overexpression of CnA increased endogenous Brn-3b, in vivo, protein levels were quantified in hearts taken from CnA transgenic mice (CnA-TG) with cardiomyocyte specific overexpression of CnA. Figure 4f(i, ii) shows that CnA-TG hearts expressed higher endogenous Brn-3b(s) protein, when compared with non-transgenic hearts indicating that high CnA expression induces Brn-3b in the heart.

### Known Brn-3b target genes that affect cell fate are increased in response to AngII

**Fig. 5:**
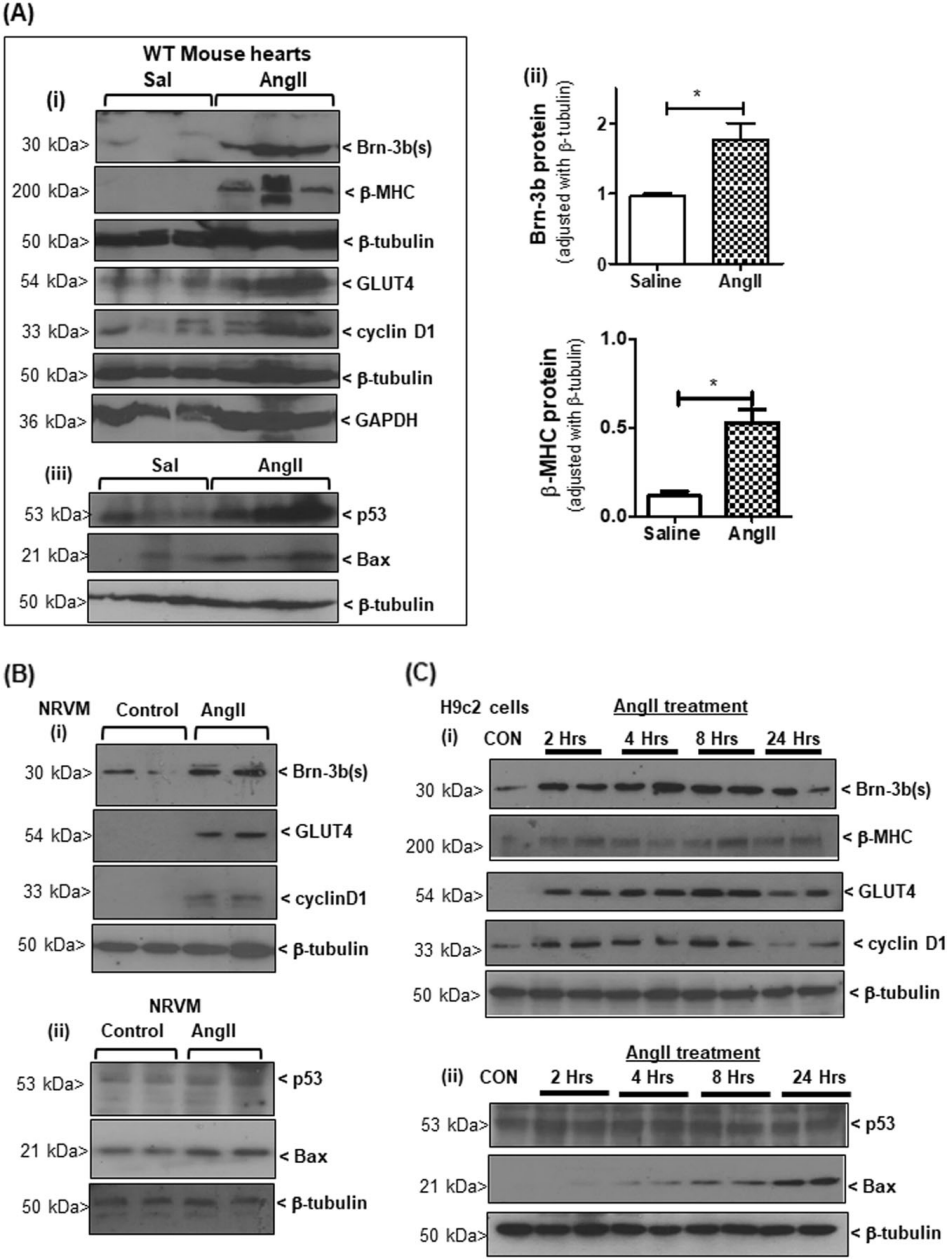
Brn-3b target genes are regulated following AngII treatment. **a** (i) Representative western blots showing changes in different proteins including Brn-3b(s) and hypertrophic marker β-MHC as well as known Brn-3b target genes GLUT4 and cyclin D1, in hearts taken from wild-type mice treated with AngII and compared with control hearts taken from age-matched control mice infused with saline (Sal). The approximate molecular weight (kD) of each protein is indicated. β-tubulin or GADPH are included to show variation in protein loading between samples. (ii) Graphical representation showing changes in Brn-3b and β-MHC proteins in AngII-treated WT hearts compared with saline controls (*n* ≥ 7). *p < 0.05, evaluated with students’ *t* test. (iii) Representative blots showing Bax and p53 proteins quantified in cellular extracts (as above). β-tubulin indicates variation in proteins loading from different samples. **b** Representative western blot showing changes in Brn-3b(s), GLUT4 and cyclin D1 protein levels in NRVM following treatment with AngII, compared with untreated controls while β-tubulin indicates variation in proteins loading from different samples. **c** Representative western blots showing Brn-3b, βMHC and Brn-3b target genes, GLUT4 and cyclin D1 in H9c2 cells, at different times after AngII treatment. (ii) Western blots showing increased pro-apoptotic Bax protein at later stages after AngII treatment

Known Brn-3b target genes, cyclin D1, GLUT4 and Bax are implicated in hypertrophic responses^17,50–52^ so we analysed for changes in expression of these genes in AngII-treated hearts that express the hypertrophic marker, βMHC and high Brn-3b (Fig. 5a(i, ii)). Representative western blots show that increased Brn-3b correlated with higher levels of cyclin D1 and GLUT4 (Fig. 5a(i)) and Bax (iii). p53 protein was also increased in AngII-treated hearts (Fig. 5a(iii)) so may cooperate with Brn-3b to maximally induced Bax.

Similar studies carried out in AngII-treated NRVM cultures show that increased Brn-3b in AngII-treated cells was associated with induction of GLUT4 and cyclin D1 (Fig. 5b(i)), when compared with untreated cells. Low levels of Bax in untreated cells suggest basal stress responses in the primary NRVM cultures but this was significantly increased following AngII treatment (Fig. 5b(ii)).

More detailed time course analyses were performed in H9C2 cells and the data shows that Brn-3b was increased, as early as 2 h after treatment, with progressive increases at later times and this correlated with induction of β−MHC (Fig. 5c). Brn-3b target genes GLUT4 and cyclin D1 were also increased by 2 h after AngII treatment, with maximal expression at 8 h. In contrast, pro-apoptotic Bax was undetectable at early stages but was induced at later stages, with maximal expression seen at 24 h (Fig. 5c (ii)). Since p53 expression did not change significantly following AngII treatment in these cells, maximal Bax induction at 24 hours seem to depend on co-expression of both Brn-3b and p53, as previously shown^40^. These results indicate that AngII induction of Brn-3b can trigger time-dependent, differential regulation of distinct target genes in cardiomyocytes and its effects will be affected by co-expression with other regulators such as p53.

### Loss of Brn-3b causes altered heart function at baseline

**Fig. 6:**
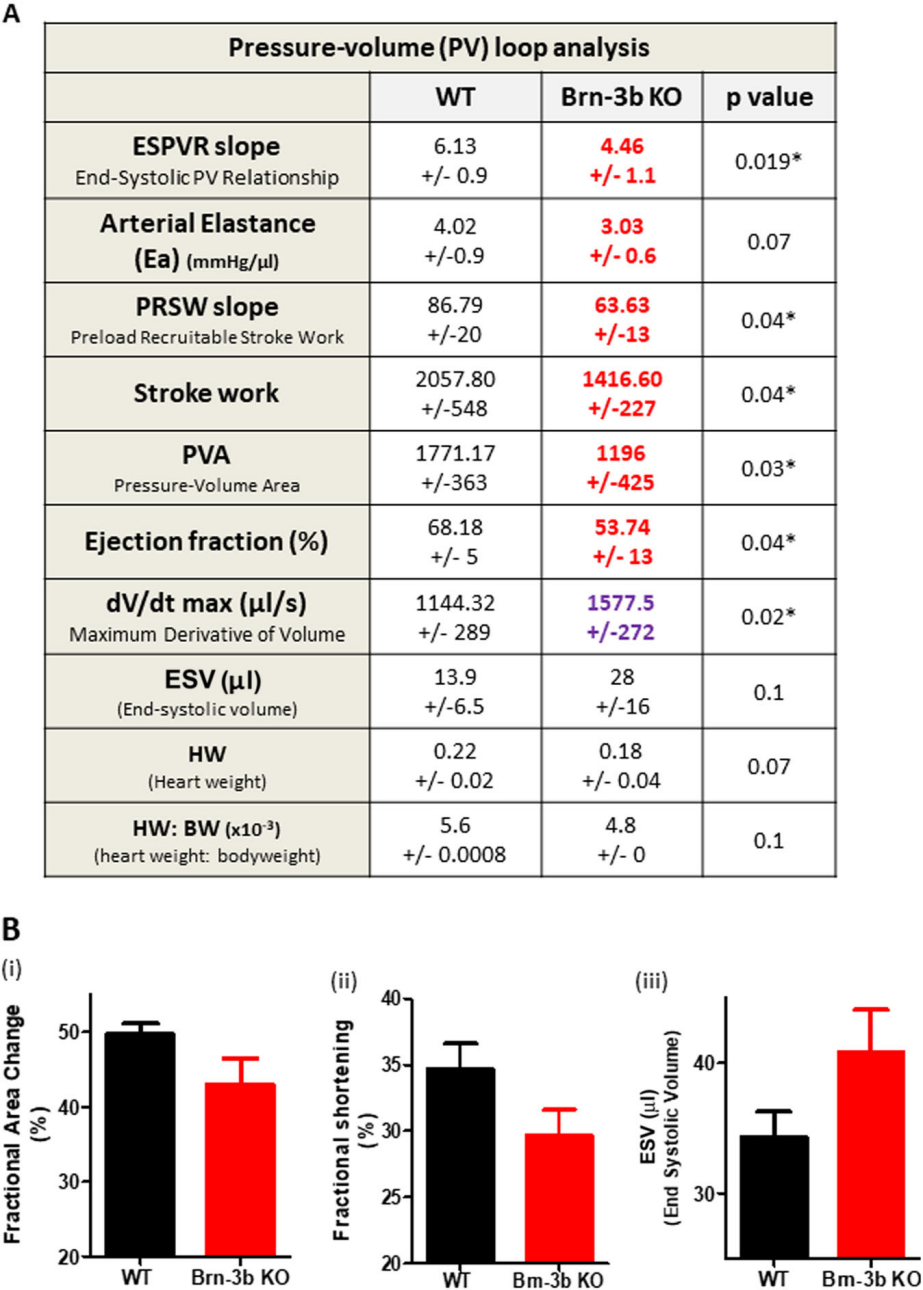
Baseline differences in Brn-3b KO heart function. **a** Summary of results from pressure–volume measurements in mouse hearts collected using the Scisense ADVantage^TM^ Admittance PV loop system, analysed with PowerLab software and statistical significance determined using students *t* test in Excel and Prism. Data represents mean and standard error (±) from six mice within each group (11 months old). *p ≤ 0.05. ESPVR = end systolic pressure–volume relationship; Ea arterial elastance; PRSW preload recruitable stroke work; PVA pressure–volume area; d*V*/d*t*_Max_ maximum conductance velocity; Ves end systolic volume; HW heart weight; HW:BW ratio heart weight:body weight ratio. **b** Echocardiography data showing parameters that are altered in Brn-3b KO mouse hearts when compared with age-matched WT control hearts. Data represent mean and standard error of multiple hearts (*n* ≥ 5)

To determine how loss of Brn-3b affected cardiac function at baseline, PV loop measurements were undertaken on available male Brn-3b KO mutants (*n* = 6; 11 months) and age-matched WT controls. Figure 6a shows a summary of key changes observed in Brn-3b KO hearts. Lower ESPVR and a trend of reduced arterial elastance, (Ea), in mutant hearts may indicate reduced contractility and this was associated with lower ejection fraction (EF); pressure–volume area (PVA); reduced stroke work (SW) and pre-load recruitable stroke work (PRSW), which suggests reduced efficiency. Similarly, mutant hearts showed that changes in d*V*/d*t*_Max_, an indicator of cardiac contractility, was associated with increased end systolic volume (ESV) (KO = 28 ml ± 16) when compared with WT controls (WT-13.9 ± 6.5), albeit not reaching significance due to large variability. Nevertheless, such findings may indicate that mutant hearts cannot empty effectively, resulting in higher ESV. Similar adverse changes in function of Brn-3b KO hearts were also demonstrated by echocardiography, which showed reduced fractional area change (FAC); fractional shortening (FS) and increased ESV, compared with WT control hearts. Therefore, echocardiography was used for subsequent longitudinal analyses of heart function, in AngII-treated and control mice.

### Attenuated hypertrophic responses in Brn-3b KO mutants following AngII treatment

**Fig. 7:**
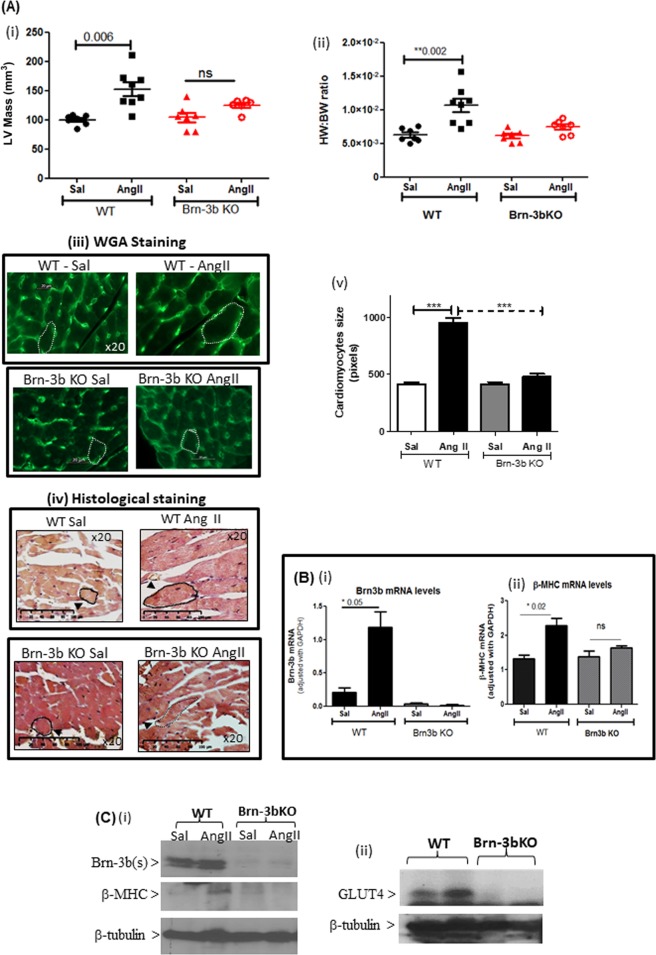
Attenuated hypertrophic responses to AngII in Brn-3b KO hearts. Echocardiography data showing changes in (i) LV mass or (ii) HW:BW ratio, in hearts from male Brn-3b KO (KO) mice and age-matched, WT control mice following AngII or saline infusion for 4 weeks. Groups of 6–8 mice were used and data represent the mean and standard error.**p < 0.01 using either students *t* test or two-way ANOVA and post-hoc Bonferroni analyses. (iii) Representative images showing changes in cardiomyocyte size in hearts taken from WT or Brn-3b KO mice, infused with either saline (Sal) or AngII and stained with wheat germ agglutinin (WGA) or (iv) histochemical staining, e.g Masson’s trichrome. Images shown at ×20 magnification. Dotted lines show representative cell surface area used for analysing differences in cardiomyocyte size in wild-type or Brn-3b KO hearts following AngII-treated and compared with appropriate saline controls (v) The mean ± SEM of cell surface area measurements taken from multiple hearts (*n* ≥ 3 independent hearts) with >30 cells analysed from each heart section. **b** Data from qRT-PCR to analyse changes in (i) Brn-3b or (ii) β−MHC mRNA in hearts taken from AngII or saline-treated WT male mice or Brn-3b KO mutants. **c** Representative western blots showing changes in protein expression, as indicated, in hearts taken from WT and Brn-3b KO mutants, treated with AngII or saline controls (Sal). β-tubulin blots show difference in total protein between samples

To study how loss of Brn-3b affected hypertrophic responses in the heart, male Brn-3b KO mice (~2 months old) and age-matched WT littermates were administered with AngII or saline (via mini osmotic pumps; up to 4 weeks). Figure 7a(i, ii) shows that AngII treatment resulted in the expected hypertrophic responses in WT hearts, i.e. increased LV mass and HW:BW ratio, when compared with saline controls. This was confirmed by analysing cardiomyocyte area in AngII-treated or control heart sections, stained to delineate cell membrane e.g. WGA (Fig. 7a(iii)) or histological stains (H + E or Masson’s trichrome) (Fig. 7a(iv)). Quantification of area change, using image J, showed significant increases in cardiomyocyte area in AngII-treated WT hearts, when compared with saline control WT hearts (Fig. 7a(v)), whereas no significant differences were observed in AngII-treated Brn-3b KO hearts either in LV mass (Fig. 6a(i)), HW:BW ratio (Fig. 7a(ii)) or cardiomyocyte area (Fig. 7a(iii–v)).

At the molecular level, increased Brn-3b expression in AngII was associated with an expected induction of βMHC in WT hearts, either at mRNA or protein levels (Fig. 7b(ii), c(ii)) but β-MHC mRNA and protein were unchanged in AngII-treated Brn-3b KO hearts (Fig. 7b(ii), c(i)). The Brn-3b target gene, GLUT4, was also reduced in AngII-treated Brn-3b KO hearts, when compared with WT controls (Fig. 6c(ii)) suggesting that the loss of such Brn-3b target genes may contribute to altered responses in the mutant hearts.

### Reduced cardiac function in AngII-treated Brn-3b KO hearts correlates with increased fibrosis and adverse remodelling

**Fig. 8:**
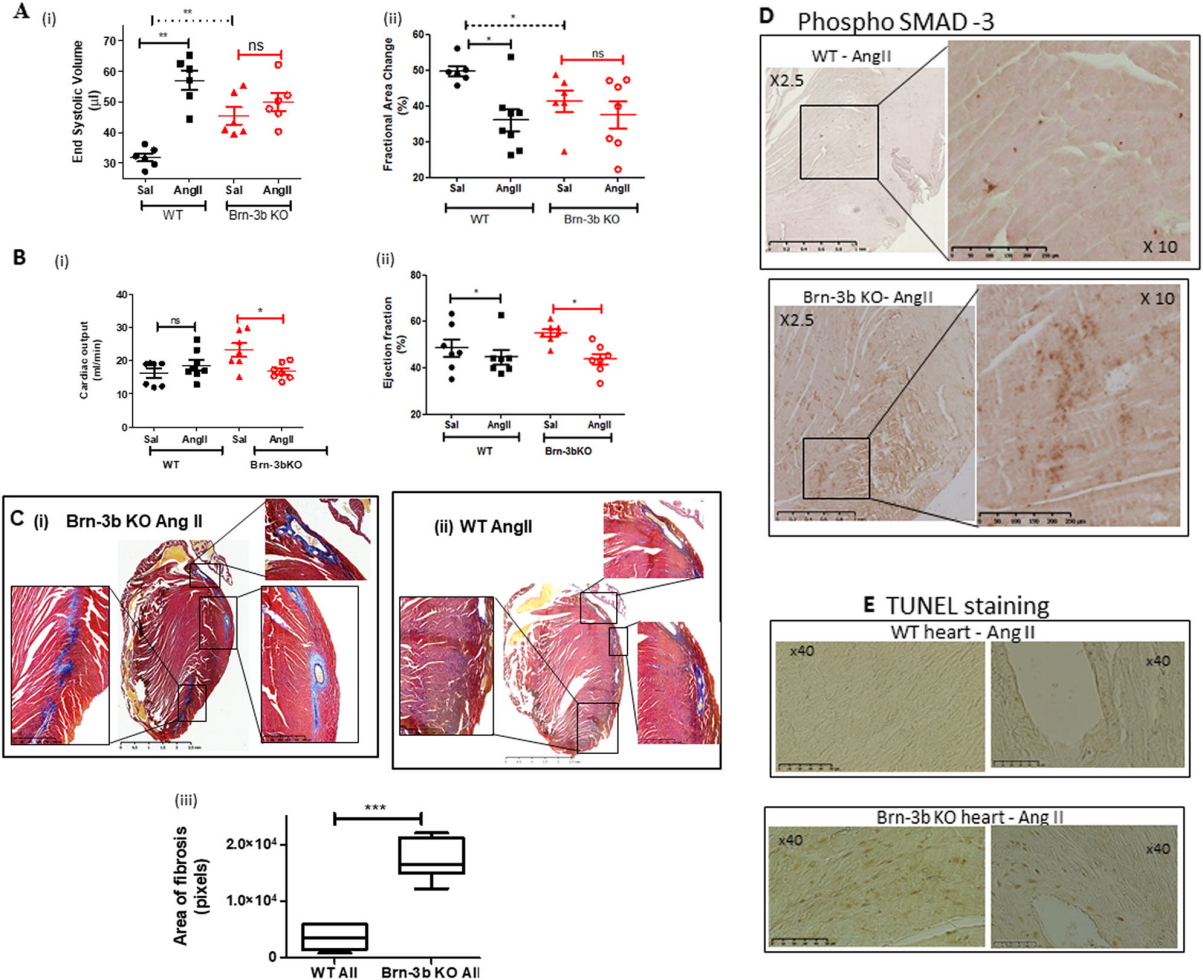
Reduced cardiac function in male Brn-3b KO hearts following AngII treatment. **a**. Echocardiography data showing changes in (i) end systolic volumes or (ii) fractional area change in Brn-3b KO hearts compared with WT control either in control saline (Sal) hearts or hearts infused with AngII (AII) for 4 weeks. Significant differences between groups are indicated by (**), as determined either using Students *t* test or two-way ANOVA and Bonferroni post-hoc test. **b** Changes in cardiac output (i) or ejection fraction (ii) in untreated control (Sal) WT or Brn-3b KO mice or treated with AngII for up to 4 weeks. Data represents the mean and standard error from 6–8 mice per group. Statistical significance between groups was determined using Students *t* test or two-way ANOVA and Bonferroni post-hoc test (*<0.05; **<0.01). **c** Representative images showing Masson’s trichrome staining of hearts sections prepared from (i) Brn-3b KO mice treated with AngII, or (ii) WT control mice treated with AngII. Images were captured using Hammamatsu Nanozoomer imaging system and shown at ×1.5–40 magnification. Red/Pink staining represent cytoplasm staining in muscle cells, dark purple/black indicates cell nuclei and blue staining indicates deposition of extracellular matrix proteins e.g. collagen. (iii) Graph representing differences in the areas with signs of fibrosis (increased ECM deposition) in WT or Brn-3b heart following AngII treatment. Areas of fibrosis were measured using image J in ≥5 independent heart sections, shown as ± SEM and significance determined using students *t* test (***p < 0.001). **d** Representative images showing anti-phospho-SMAD 3 antibody DAB immunostaining in sections of hearts taken from AngII-treated mice as indicated [WT mice (top panel) or Brn-3b KO mice (lower panel)]. Positive cells are stained brown in images shown either at lower magnification (×2.5) to highlight the extent of increased SMAD protein expression in AngII-treated Brn-3b KO hearts or at higher magnification (×10) displays the intensity of staining. **e** Representative TUNEL staining in heart sections taken from AngII-treated WT mice (top panel) or Brn-3b KO mice (lower panel). TUNEL positive cells appear brown in the images (shown at ×40 magnification)

Echocardiography analysis of AngII treated and control hearts also revealed subtle but clear differences in cardiac function of Brn-3b KO when compared with WT hearts, either at baseline or in response to AngII (Fig. 8a, b). Thus, Brn-3b KO mutants displayed higher high ESV at baseline [a(i)] and reduced FAC, which were associated with increased cardiac output (CO) (b). Moreover, AngII treatment caused significant reduction in CO and EF but sustained high ESV in Brn-3b KO mice, which may indicate failure of the mutant heart to cope with stress.

Masson’s Trichrome staining of experimental hearts identified structural changes in AngII-treated Brn-3b KO (Fig. 8c) with evidence for increased extracellular matrix deposition and fibrosis (blue), particularly in the left ventricle and around blood vessels, when compared with AngII-treated WT hearts. Quantification of the fibrotic areas showed significantly more fibrosis in Brn-3b KO hearts when compared with WT controls (Fig. 8c(iii)). Analysis for expression of fibrotic marker, phospho-SMAD, showed significantly increased staining in AngII-treated Brn-3b KO hearts (lower panel, Fig. 8d) when compared with appropriate WT hearts (top panel). TUNEL staining to analyse for cell death also showed increased staining in AngII-treated Brn-3b KO when compared with AngII-treated WT hearts (Fig. 7e). These findings suggest that adverse remodelling in Brn-3b KO hearts is associated with increased cell death, which may contribute to dilatation and reduced contractile function seen by echocardiography.

## Discussion

Terminally differentiated cardiomyocytes in adult hearts cannot proliferate readily in response to chronic cardiac stressors so adapt by undergoing hypertrophic growth to maintain cardiac output and function. Characteristic adaptive changes such as sarcomeric reorganisation and metabolic switching, arise because of extensive reprogramming of gene expression^2,53^, which includes re-expression of foetal genes (e.g. β−MHC and ANP), cell cycle genes (e.g. cyclin D1/CDK4) and oncogenes (e.g. c-fos or c-myc)^18^ or increased expression of GLUT4 glucose transporter^20,54^. In contrast, sustained stress which induce pro-apoptotic genes e.g. p53 and Bax, can contribute to irreversible cardiomyocyte death, adverse remodelling and subsequent progression to heart failure^25^. Yet, master regulators that control the transition between early adaptive responses and pathological events are not fully understood^1,53^. Previous studies have shown that the Brn-3b TF is re-expressed in injured cardiomyocytes and can alter cell fate^40,43^ and in this study we show that Brn-3b is induced in response to hypertrophic stress and may have potentially important roles in controlling hypertrophic responses in the heart.

Brn-3b mRNA and protein are induced by AngII treatment in WT mouse hearts that undergo characteristic hypertrophic changes including increased LV mass and HW:BW ratio and re-expression of hypertrophic genes e.g. βMHC, ANP^55^. Since AngII treatment induces Brn-3b mRNA and protein in isolated NRVM that undergo hypertrophy^48^, we conclude that Brn-3b induction in cardiomyocytes will be important for hypertrophic responses in the heart. Similarly, AngII stimulates Brn-3b mRNA and protein in hypertrophic H9c2 cells expressing β-MHC and displaying characteristic sarcomeric remodelling. Based on these findings and data from previous studies^40^, we determined that the rat heart-derived H9c2 cell line provides a useful adjunct for studying the effects of Brn-3b in cardiomyocytes^40,42^.

At the protein level, AngII treatment causes preferential increase in the Brn-3b(s) isoform and while the roles for the distinct Brn-3b isoforms are still unclear, high levels of Brn-3b(s) protein is strongly associated with increased proliferative and oncogenic potential cancer cells^34,35^. This is interesting because re-expression of foetal oncogenes is a characteristic feature of cardiac hypertrophy and may represent an adaptive response to chronic stress^18,56^. However, whereas oncogenes such as c-fos and c-myc display immediate-early (IE) expression profiles in hypertrophic hearts (30–60 min, respectively)^18,56^, Brn-3b expression is sustained in for up to 24 h and may therefore have as yet unknown roles in the stressed heart.

Importantly, Brn-3b activation in hypertrophic cardiomyocytes occurs via different, well-characterised hypertrophic signalling pathways including p42/44 MAPK and CnA, suggesting that it may be a central mediator of hypertrophic responses in cardiomyocytes. The effects of AngII on the Brn-3b promoter are mediated via the p42/44 MAPK pathway and are blocked by the MEK1/2 inhibitor, PD98059 but not by p38 MAPK inhibitor (SB203580)^56–59^. This is consistent with previous studies showing that p42/44 MAPK pathway is important for activation of the Brn-3b promoter in human breast cancer cells^47^.

Brn-3b activation by CnA, is also highly relevant because of the central role of this pathway in driving hypertrophic responses in the heart^11^. Furthermore, higher endogenous Brn-3b levels in hearts from cardiac-specific CnA transgenic mice^13^ indicate that this TF is a downstream target of CnA. This is further strengthened by the presence of multiple NFAT binding sites in the proximal Brn-3b promoter and ChIP assay data showing that NFAT protein is, indeed, bound to the Brn-3b promoter, in vivo^13,14^.

Interestingly, there is an overlap between AngII and CnA pathways in regulating Brn-3b expression because AngII treatment increases NFAT binding to the promoter whereas the CnA inhibitor, ascomicin, blocks AngII induction of Brn-3b. Crosstalk between MAPK/ERK and CnA pathways have been reported previously since AngII can directly activate the CnA pathway^49^, whereas ERK1/2 phosphorylation of an N-terminal serine residue in NFAT can enhance transcriptional activity on its target genes^11,14^. CnA may also be activated indirectly by MEK5/ERK5 (BMK)^60^, which phosphorylates and inhibits the negative regulator, MCIP1 following AngII stimulation^61^. This data suggests that canonical hypertrophic mediators such as AngII and CnA converge to activate Brn-3b in cardiomyocytes so this master regulator is likely to play a central role in hypertrophic responses in stressed hearts.

As a TF, Brn-3b(s) regulates multiple target genes and thereby drive complex cellular effects. Here we show that known Brn-3b target genes, cyclin D1^34^, GLUT4^30^ and Bax^40,41^, are increased by AngII in intact hearts, isolated NRVM cultures or H9c2 cells. Time-course studies identify differential expression patterns of Brn-3b target genes following AngII treatment with maximal expression of cyclin D1 and GLUT4 seen at earlier time points when pro-apoptotic Bax expression is low but are reduced by 24 h, when Bax is maximally induced. Although different Brn-3b target genes are implicated in hypertrophic responses, each is associated with distinct cellular effects. For example, cyclin D1 KO mice display attenuated hypertrophy, whereas transgenic mice over-expressing cyclin D1 develop enlarged hearts^16,17^. Similarly, GLUT4, is increased in hypertrophic hearts^30^ and may contribute to metabolic switching, from fatty acid to glucose metabolism in stressed hearts^54^. In contrast, induction of Bax and p53 are linked to cardiomyocyte apoptosis in failing hearts^25,62,63^. We found low p53 levels in normal hearts but increased expression in AngII-treated hypertrophic hearts. However, cell culture models, NRVM and H9C2, express higher basal p53 levels, which may reflect apoptosis associated with increased cell turnover. Although p53 can stimulate Bax, maximal activation in NRVM requires co-expression of Brn-3b and p53 since siRNA to reduce Brn-3b can block Bax induction despite sustained p53 expression. Therefore, Brn-3b may regulate distinct target genes in hypertrophic cardiomyocytes at early stages when p53 expression is low but, at later stages, when p53 is increased, it may cooperate with p53 to drive apoptotic effects associated with pathological changes.

Analyses of heart function in male Brn-3b KO mice have also identified key baseline differences between mutant and WT control hearts, which confirms essential functions for this TF in controlling cardiac function. PV loop analyses carried out on 11-month-old male mice show contractile dysfunction in Brn-3b KO mutants which is reflected in reduced ESPVR, Ea, EF and reduced capacity for work, i.e. reduced SW, PRSW and PVA. Increased d*V*/d*t*_Max_ and increased ESV suggests that mutant hearts are less effective at emptying at end systole, when compared with age match WT controls. Brn-3b KO mutants also show clear trends towards lower HW and HW/BW ratio but high variability meant this did not reach statistical significance so will have to be confirmed in larger studies. Contractile dysfunctions in male Brn-3b KO hearts are also detected using echocardiography e.g. reduced FAC and FS and increased ESV. Since this technique offers advantages for undertaking longitudinal studies over PV loop studies, which is normally done as terminal procedure, then echocardiography was used to analyse heart function in many later studies.

Importantly, hearts from Brn-3b KO mice display blunted response to AngII treatment, which induces the expected hypertrophic changes in WT male hearts. Furthermore, adverse functional and contractile changes in mutant hearts at baseline, are worsened following AngII treatment with poor contractile function linked to reduced CO and EF and increased ESV. There is also evidence for extensive fibrosis and remodelling in AngII-treated Brn-3b KO hearts, particularly in left ventricle and around blood vessels of AngII-treated male which correlates with increased phospho-SMAD-3, a marker of pathological remodelling in stressed hearts. As a downstream effector of TGF beta pathway, phospho-SMAD 3, is implicated in pathological responses to chronic stress in murine hearts^64^, so increased phospho-SMAD 3 indicates maladaptive responses in AngII-treated Brn-3b KO hearts. Similarly, increased cardiomyocyte death, detected by TUNEL staining in AngII-treated Brn-3b KO hearts, suggest failure of mutant hearts to adapt to stress, which may result in progression to heart failure.

Further analysis of mutant hearts and WT controls for changes in known Brn-3b target genes associated with hypertrophic responses suggest complex roles for this regulator in controlling cardiomyocytes fate. For example, although Brn-3b can cooperate with p53 to induce pro-apoptotic Bax, AngII-treated Brn-3b KO hearts show evidence for increased cardiomyocyte death, thus implicating Brn-3b in other early responses required for adaptation and survival of these cells. Furthermore, GLUT4, which is an insulin-responsive transporter associated with metabolic switching from fatty acid to glucose metabolism, is significantly reduced in AngII-treated Brn-3b KO hearts, so may represent an important Brn-3b target gene required for in mediating hypertrophic responses in the heart. However, other as yet unknown, cardiac-specific target genes regulated by Brn-3b, may also play important roles in this process^51,65^. Thus, Brn-3b may represent a novel regulator that is required to control adaptive hypertrophic responses in male heart, in response to stress.

## References

[1] Frey N, Olson EN (2003). Cardiac hypertrophy: the good, the bad, and the ugly. Annu. Rev. Physiol..

[2] Komuro I (2001). Molecular mechanism of cardiac hypertrophy and development. Jpn. Circ. J..

[3] Richey PA, Brown SP (1998). Pathological versus physiological left ventricular hypertrophy: a review. J. Sports Sci..

[4] Yazaki Y, Tsuchimochi H, Kurabayashi M, Komuro I (1989). Molecular adaptation to pressure overload in human and rat hearts. J. Mol. Cell Cardiol..

[5] Anversa P, Kajstura J, Olivetti G (1996). Myocyte death in heart failure. Curr. Opin. Cardiol..

[6] Molkentin JD, Dorn GW (2001). Cytoplasmic signaling pathways that regulate cardiac hypertrophy. Annu. Rev. Physiol..

[7] Shimoyama M (1999). Calcineurin plays a critical role in pressure overload-induced cardiac hypertrophy. Circulation.

[8] Wilkins BJ, Molkentin JD (2004). Calcium-calcineurin signaling in the regulation of cardiac hypertrophy. Biochem. Biophys. Res. Commun..

[9] Wilkins BJ (2004). Calcineurin/NFAT coupling participates in pathological, but not physiological, cardiac hypertrophy. Circ. Res..

[10] Yamazaki T, Yazaki Y (2000). Molecular basis of cardiac hypertrophy. Z. Kardiol..

[11] Heineke J, Molkentin JD (2006). Regulation of cardiac hypertrophy by intracellular signalling pathways. Nat. Rev. Mol. Cell Biol..

[12] Fahmi A (2013). p42/p44-MAPK and PI3K are sufficient for IL-6 family cytokines/gp130 to signal to hypertrophy and survival in cardiomyocytes in the absence of JAK/STAT activation. Cell Signal..

[13] Molkentin JD (2004). Calcineurin-NFAT signaling regulates the cardiac hypertrophic response in coordination with the MAPKs. Cardiovasc. Res..

[14] Molkentin JD (1998). A calcineurin-dependent transcriptional pathway for cardiac hypertrophy. Cell.

[15] Busk PK (2005). Cyclin D2 induces proliferation of cardiac myocytes and represses hypertrophy. Exp. Cell Res..

[16] Busk PK (2002). Involvement of cyclin D activity in left ventricle hypertrophy in vivo and in vitro. Cardiovasc. Res..

[17] Tamamori-Adachi M (2002). Expression of cyclin D1 and CDK4 causes hypertrophic growth of cardiomyocytes in culture: a possible implication for cardiac hypertrophy. Biochem. Biophys. Res. Commun..

[18] Komuro I (1990). Stretching cardiac myocytes stimulates protooncogene expression. J. Biol. Chem..

[19] Kudoh S (1997). Angiotensin II stimulates c-Jun NH2-terminal kinase in cultured cardiac myocytes of neonatal rats. Circ. Res..

[20] Abel ED (1999). Cardiac hypertrophy with preserved contractile function after selective deletion of GLUT4 from the heart. J. Clin. Investig..

[21] Leri A (2003). Ablation of telomerase and telomere loss leads to cardiac dilatation and heart failure associated with p53 upregulation. EMBO J..

[22] Mandl A, Huong PL, Toth K, Zambetti G, Erhardt P (2011). Puma deletion delays cardiac dysfunction in murine heart failure models through attenuation of apoptosis. Circulation.

[23] Sano M (2007). p53-induced inhibition of Hif-1 causes cardiac dysfunction during pressure overload. Nature.

[24] Del Re,DP, Miyamoto S, Brown JH (2007). RhoA/Rho kinase up-regulate Bax to activate a mitochondrial death pathway and induce cardiomyocyte apoptosis. J. Biol. Chem..

[25] Condorelli G (1999). Increased cardiomyocyte apoptosis and changes in proapoptotic and antiapoptotic genes bax and bcl-2 during left ventricular adaptations to chronic pressure overload in the rat. Circulation.

[26] Latchman, D. *Gene Control*. 2nd edn (Taylor and Francis Group, Milton Park, Abingdon, UK, 2015).

[27] Akazawa H, Komuro I (2003). Roles of cardiac transcription factors in cardiac hypertrophy. Circ. Res..

[28] van Berlo JH (2010). The transcription factor GATA-6 regulates pathological cardiac hypertrophy. Circ. Res..

[29] Budhram-Mahadeo VS, Latchman DS (2006). Targeting Brn-3b in breast cancer therapy. Expert. Opin. Ther. Targets.

[30] Bitsi S (2016). Profound hyperglycemia in knockout mutant mice identifies novel function for POU4F2/Brn-3b in regulating metabolic processes. Am. J. Physiol. Endocrinol. Metab..

[31] Budhram-Mahadeo V, Ndisang D, Ward T, Weber BL, Latchman DS (1999). The Brn-3b POU family transcription factor represses expression of the BRCA-1 anti-oncogene in breast cancer cells. Oncogene.

[32] Gan L, Wang SW, Huang Z, Klein WH (1999). POU domain factor Brn-3b is essential for retinal ganglion cell differentiation and survival but not for initial cell fate specification. Dev. Biol..

[33] Pan L, Deng M, Xie X, Gan L (2008). ISL1 and BRN3B co-regulate the differentiation of murine retinal ganglion cells. Development.

[34] Budhram-Mahadeo VS (2008). Proliferation-associated Brn-3b transcription factor can activate cyclin D1 expression in neuroblastoma and breast cancer cells. Oncogene.

[35] Irshad S, Pedley RB, Anderson J, Latchman DS, Budhram-Mahadeo V (2004). The Brn-3b transcription factor regulates the growth, behavior, and invasiveness of human neuroblastoma cells in vitro and in vivo. J. Biol. Chem..

[36] Dennis JH, Budhram-Mahadeo V, Latchman DS (2001). The Brn-3b POU family transcription factor regulates the cellular growth, proliferation, and anchorage dependence of MCF7 human breast cancer cells. Oncogene.

[37] Samady L, Dennis J, Budhram-Mahadeo V, Latchman DS (2004). Activation of CDK4 gene expression in human breast cancer cells by the Brn-3b POU family transcription factor. Cancer Biol. Ther..

[38] Lee SA (2005). Expression of the Brn-3b transcription factor correlates with expression of HSP-27 in breast cancer biopsies and is required for maximal activation of the HSP-27 promoter. Cancer Res..

[39] Samady L (2006). TheBrn-3b POU family transcription factor represses plakoglobin gene expression in human breast cancer cells. Int. J. Cancer.

[40] Budhram-Mahadeo V, Fujita R, Bitsi S, Sicard P, Heads R (2014). Co-expression of POU4F2/Brn-3b with p53 may be important for controlling expression of pro-apoptotic genes in cardiomyocytes following ischaemic/hypoxic insults. Cell Death Dis..

[41] Budhram-Mahadeo VS (2006). Brn-3b enhances the pro-apoptotic effects of p53 but not its induction of cell cycle arrest by cooperating in trans-activation of bax expression. Nucleic Acids Res..

[42] Maskell LJ (2017). Essential but partially redundant roles for POU4F1/Brn-3a and POU4F2/Brn-3b transcription factors in the developing heart. Cell Death Dis..

[43] Farooqui-Kabir SR (2008). Cardiac expression of Brn-3a and Brn-3b POU transcription factors and regulation of Hsp27 gene expression. Cell Stress Chaperones.

[44] Stuckey DJ, Carr CA, Tyler DJ, Clarke K (2008). Cine-MRI versus two-dimensional echocardiography to measure in vivo left ventricular function in rat heart. NMR Biomed..

[45] Clark James E., Marber Michael S. (2012). Advancements in pressure-volume catheter technology - stress remodelling after infarction. Experimental Physiology.

[46] Clark JE, Kottam A, Motterlini R, Marber MS (2009). Measuring left ventricular function in the normal, infarcted and CORM-3-preconditioned mouse heart using complex admittance-derived pressure volume loops. J. Pharmacol. Toxicol. Methods.

[47] Ounzain S (2011). Proliferation-associated POU4F2/Brn-3b transcription factor expression is regulated by oestrogen through ERalpha and growth factors via MAPK pathway. Breast Cancer Res..

[48] Watkins SJ, Borthwick GM, Arthur HM (2011). The H9C2 cell line and primary neonatal cardiomyocyte cells show similar hypertrophic responses in vitro. Vitr. Cell Dev. Biol. Anim..

[49] Wang LN (2008). Involvement of calcium-sensing receptor in cardiac hypertrophy-induced by angiotensinII through calcineurin pathway in cultured neonatal rat cardiomyocytes. Biochem. Biophys. Res. Commun..

[50] Stuck BJ, Lenski M, Bohm M, Laufs U (2008). Metabolic switch and hypertrophy of cardiomyocytes following treatment with angiotensin II are prevented by AMP-activated protein kinase. J. Biol. Chem..

[51] Szablewski L (2017). Glucose transporters in healthy heart and in cardiac disease. Int. J. Cardiol..

[52] Hotchkiss A (2012). Role of D-type cyclins in heart development and disease. Can. J. Physiol. Pharmacol..

[53] Ahuja P, Sdek P, MacLellan WR (2007). Cardiac myocyte cell cycle control in development, disease, and regeneration. Physiol. Rev..

[54] Frey N, Olson EN (2002). Modulating cardiac hypertrophy by manipulating myocardial lipid metabolism?. Circulation.

[55] Fernandez-Alfonso MS, Ganten D, Paul M (1992). Mechanisms of cardiac growth. The role of the renin-angiotensin system. Basic Res. Cardiol..

[56] Izumo S, Nadal-Ginard B, Mahdavi V (1988). Protooncogene induction and reprogramming of cardiac gene expression produced by pressure overload. Proc. Natl Acad. Sci. USA.

[57] Braz JC (2003). Targeted inhibition of p38 MAPK promotes hypertrophic cardiomyopathy through upregulation of calcineurin-NFAT signaling. J. Clin. Investig.

[58] Bueno OF (2000). The MEK1-ERK1/2 signaling pathway promotes compensated cardiac hypertrophy in transgenic mice. EMBO J..

[59] Pellieux C, Sauthier T, Aubert JF, Brunner HR, Pedrazzini T (2000). Angiotensin II-induced cardiac hypertrophy is associated with different mitogen-activated protein kinase activation in normotensive and hypertensive mice. J. Hypertens..

[60] Suzaki Y (2002). Hydrogen peroxide stimulates c-Src-mediated big mitogen-activated protein kinase 1 (BMK1) and the MEF2C signaling pathway in PC12 cells: potential role in cell survival following oxidative insults. J. Biol. Chem..

[61] Abbasi S (2006). Protein kinase-mediated regulation of calcineurin through the phosphorylation of modulatory calcineurin-interacting protein 1. J. Biol. Chem..

[62] Ikeda S, Hamada M, Hiwada K (1999). Cardiomyocyte apoptosis with enhanced expression of P53 and Bax in right ventricle after pulmonary arterial banding. Life Sci..

[63] Chatterjee Arunachal, Mir Saiful A., Dutta Dipanjan, Mitra Arkadeep, Pathak Kanchan, Sarkar Sagartirtha (2011). Analysis of p53 and NF-κB signaling in modulating the cardiomyocyte fate during hypertrophy. Journal of Cellular Physiology.

[64] Koitabashi N (2011). Pivotal role of cardiomyocyte TGF-beta signaling in the murine pathological response to sustained pressure overload. J. Clin. Investig..

[65] Wende AR (2017). Glucose transporter 4-deficient hearts develop maladaptive hypertrophy in response to physiological or pathological stresses. Am. J. Physiol. Heart Circ. Physiol..

